# Expression of free radicals by peritoneal cells of sheep during the early stages of *Fasciola hepatica* infection

**DOI:** 10.1186/s13071-018-3072-5

**Published:** 2018-09-06

**Authors:** Raúl Pérez-Caballero, Leandro Buffoni, F. Javier Martínez-Moreno, Rafael Zafra, Verónica Molina-Hernández, José Pérez, Álvaro Martínez-Moreno

**Affiliations:** 1 Animal Health Department (Parasitology and Parasitic Diseases), Faculty of Veterinary Medicine, University of Córdoba, Campus de Rabanales, Ctra. Madrid-Cádiz, km 396, 14014 Córdoba, Spain; 2 Anatomy and Comparative Pathology Department, Faculty of Veterinary Medicine, University of Córdoba, Campus de Rabanales, Ctra. Madrid-Cádiz, km 396, 14014 Córdoba, Spain

**Keywords:** *Fasciola hepatica*, Nitric oxide, ROS, Peritoneal cells, Vaccines, Sheep

## Abstract

**Background:**

The majority of vaccination studies against infection with *F. hepatica* in a natural host have been conducted at the late stage of the infection when the host's immune response is already immunomodulated by the parasite towards a Th2 non-protective response. This study was aimed at analysing the dynamic of the cell populations present in peritoneal liquid and the production of free radicals by the peritoneal leukocytes in infected and vaccinated sheep with recombinant cathepsin L1 of *F. hepatica* (rFhCL1) in early stages of the infection.

**Methods:**

Forty-five sheep were divided into three groups: Group 1 remained as negative control (*n* = 5), Group 2 (*n* = 20) was challenged with *F. hepatica* and Group 3 (*n* = 20) was vaccinated with rFhCL1 and challenged with *F. hepatica*. After the slaughtering, peritoneal lavages were carried out at 1, 3, 9 and 18 days post-infection (dpi) to isolate peritoneal cell populations. Flow cytometry was conducted to assess levels of hydrogen peroxide (H_2_O_2_) and nitric oxide (NO).

**Results:**

There was a significant increase in the total number of leukocytes at 9 and 18 dpi in infected and vaccinated groups. Production of H_2_O_2_ was significantly increased in peritoneal granulocytes in both infected and vaccinated groups. Production of nitric oxide showed a significant rise in the granulocytes and monocytes/macrophages in infected and vaccinated sheep. The NO production by granulocytes at 3 and 9 dpi was significantly higher in the vaccinated than in the infected animals.

**Conclusions:**

Experimental infection induced an increase in the total number of leukocytes within the abdominal cavity at 9 and 18 dpi, being more noticeable in vaccinated animals. Production of H_2_O_2_ occurred mainly in granulocytes of vaccinated and infected animals. Production of NO was incremented in vaccinated and non-vaccinated animals in all peritoneal cells. Vaccinated animals produced significant higher level of H_2_O_2_ and NO than infected animals.

## Background

Fasciolosis, caused by *Fasciola hepatica*, is a globally distributed parasitic disease that mainly affects ruminant livestock and causes great impact in terms of economic losses to the agricultural industry [1, 2]. The World Health Organization (WHO) recognises it as a food-borne trematode infection and as an important zoonotic disease. Immature and mature forms of the parasite inhabits the liver of the host and produces a hepatitis which may alter the liver function [3].

There are few effective strategies to control the disease though it is widely accepted that the use of anthelmintics is the best means to control the infection. Specific drugs differ in their efficacy as some of them may not affect early immature stages of the parasite, hence triclabendazole (TCBZ) has become the drug of choice in many countries. A key drawback lies on the anthelmintic resistance to various drugs including TCBZ which has been globally reported [4–7]. Consequently, in the past two decades, many attempts have been conducted to develop a viable vaccine showing diverse results in cattle and sheep. The majority of the vaccination studies have been focussed on the late stage of the infection when the immune response of the host is known to be already immunomodulated by the parasite towards a non-protective Th2 response and the inhibition of protective pro-inflammatory products [8, 9]. Therefore, studies in infected and vaccinated animals during the early stage of infection, when the parasite is migrating and establishing in the liver may shed light on the initial immunological pathways of the disease.

Previous studies have shown that some parasites can trigger free radical production by leukocytes including superoxide radical, nitric oxide, and hydrogen peroxide [10, 11]. In *F. hepatica* infections, resident peritoneal leukocytes such as macrophages might be involved in the killing of newly excysted juvenile liver flukes (NEJ). This may be accomplished by a parasite-specific antibody dependent mechanisms since most juvenile parasites are killed in the gut or the abdominal cavity, before reaching the liver [12]. In this way, some studies have shown an increase in the production of reactive oxygen species (ROS) produced by peritoneal leukocytes in rats infected with *Fasciola hepatica* [13, 14], which might be involved in the killing of migrating immature liver flukes during the early host-parasite interface. Moreover, free radical-induced cytotoxicity against NEJ of *Fasciola* sp. has been previously reported in infected sheep [15].

The aim of this work was to develop an *in vivo* study using flow cytometry in order to investigate the dynamic of the cell populations present in peritoneal liquid and the production of free radicals by leukocytes (macrophages and granulocytes) present in peritoneal liquid in infected animals (vaccinated and non-vaccinated) with recombinant cathepsin L1 of *F. hepatica* (rFhCL1) in early stages of the disease.

## Methods

### Animals and experimental design

Forty-five six-month-old male merino sheep obtained from a liver fluke-free farm were used for the experimental trial. Before beginning the study, animals were confirmed to be free of liver fluke infection by faecal analyses and ELISA for *F. hepatica* specific antibodies. Sheep were housed in covered pens and fed daily with hay and commercial pelleted ration.

Sheep were randomly divided into three groups: Group 1 consisted of 5 animals which were neither immunised nor experimentally challenged (*n* = 5), hence remained as negative control group, Group 2 consisted of 20 animals (*n* = 20) which were experimentally infected with *F. hepatica* (positive control group) and Group 3 consisted of 20 sheep which were immunised with rFhCL1 and experimentally challenged with *F. hepatica* (*n* = 20). In addition, animals from Groups 2 and 3 were subdivided into smaller groups of five animals each according to slaughtering day: 1, 3, 9 and 18 days post-infection (dpi).

Animals from Group 3 received the vaccine twice by subcutaneous inoculation on weeks 0 and 4 of the trial. At week 8 of the experiment, animals from Groups 2 and 3 were orally challenged with one single dose of 150 metacercariae of *F. hepatica* (Ridgeway Research Ltd., St Briavels, UK) administered in gelatine capsules using a dosing gun. As previously mentioned, five animals from Groups 2 and 3 were euthanised by an intravenous injection of T61® (Intervet, Barcelona, Spain) at each time-point; animals from the negative control group (Group 1) were euthanised on the 12th week of the trial.

### Purification of recombinant *F. hepatica* cathepsin L1

Recombinant *F. hepatica* cathepsin L1 (rFhCL1) was expressed in the yeast *Pichia pastoris* and purified as described elsewhere [16]. Yeast transformants were cultured in 250 ml BMGY broth, buffered to pH 6.0, in 1 l baffled flasks at 30 °C until an OD_600_ of 2–6 was reached. Cells were harvested by centrifugation at 2000× *g* for 5 min and protein expression was induced by resuspending the cells in 50 ml BMMY broth, buffered at pH 6.0, 7.0 or 8.0, containing 1% methanol. The cultures were grown at 30 °C with shaking at 225× *rpm* for 3 days, and filter-sterilized methanol was added daily to maintain a final concentration of 1%. Recombinant proteins were purified from the yeast medium by affinity chromatography using Ni-NTA-agarose (Qiagen, Montreal, Canada). Briefly, a column prepared with 1 ml of resin was equilibrated by passing through 10 ml 50 mM sodium phosphate buffer (pH 8.0) containing 300 mM NaCl and 10 mM imidazole. Ten millilitres of yeast media supernatant was mixed with 40 ml of the same buffer and applied to the column. The column was washed with 15 ml of 50 mM sodium phosphate buffer (pH 8.0) containing 300 mM NaCl and 20 mM imidazole, and bound protein was eluted using 50 mM sodium phosphate buffer (pH 7.0) containing 300 mM NaCl and 250 mM imidazole. Purified recombinant proteases were dialysed against phosphate buffered saline (PBS) and stored at -20 °C. Before immunisation, electrophoresis in polyacrylamide gel was carried out to check protein purity.

### Vaccine preparation

The recombinant protein cathepsin L1 of *F. hepatica* (rFhCL1) was diluted in ISA 70 Montanide adjuvant. Each immunisation dose was prepared as follows: 100 μg of rFhCL1 was diluted in PBS containing 1 mg/ml of the adjuvant, reaching a final volume of 1 ml per dose.

### Liver pathology

Necropsy was performed and the liver was removed and photographed on both visceral and diaphragmatic surface for gross evaluation. Liver tissue samples showing hepatic lesions were collected and fixed in 10% neutral buffered formalin for 24 h, then routinely processed and embedded in paraffin wax. Four-micron-thick tissue sections were stained with hematoxylin and eosin (H&E) for histopathology. Gross hepatic lesions during the early stages of infection in challenged animals (Groups 2 and 3) were counted using the Image-Pro Plus 4.0 software (Media Cybernetics, Silver Spring, MD, USA).

### Antibody detection

Blood samples were taken at weeks 0 and 6 of the trial and at 1, 3, 9 and 18 days post-infection (dpi), and plasma was collected to detect specific IgG1 and IgG2 antibodies against rFhCL1 by ELISA. Briefly, 96-well ELISA plates were coated with 5 μg/ml of rFhCL1 (100 μl/well) diluted in 0.05 M carbonate-bicarbonate buffer pH 9.6 and incubated at 37 °C overnight. After 5 washes with phosphate buffer saline (PBS) 0.05% Tween 20, plates were blocked with 100 μl/well of blocking buffer containing 1% BSA diluted in PBS and incubated at 37 °C for 30 min. To detect IgG1, wells were washed and 100 μl/well of plasma diluted in blocking buffer was added and incubated at 37 °C for 30 min. Triple serial dilutions were performed to determine endpoint titre. Similarly, IgG2 was detected by adding 100 μl/well of plasma diluted at 1:25. After washing, 100 μl/well of primary antibody diluted 1:5000 (mouse anti-bovine IgG1 and anti-bovine IgG2; 7500820–7500830 Cedi-Diagnostics, Lelystad, The Netherlands), in blocking buffer was added and incubated at 37 °C for 30 min. After incubation, wells were washed and anti-mouse IgG-HRP (STAR13B, BIO-RAD - formerly AbD-Serotec-, Kidlington, UK) was added at 37 °C for 30 min. Plates were washed and 100 μl/well of tetramethylbenzidine (TMB; Sigma-Aldrich, Madrid, Spain) were added and incubated for 10 min at room temperature. The reaction was stopped by adding of 100 μl/well of 1 M sulphuric acid and optical density was measured at 450 nm using a microplate photometer (Multiskan^TM^ FC, Thermo Fisher Scientific, Madrid, Spain). Results are shown as antibody titre -log_10_-for IgG1, and as optical density for IgG2.

### Isolation of peritoneal cell population

To collect peritoneal cell population, abdominal lavage of each sheep was immediately conducted after the slaughtering as previously described [17]. Briefly, the ventral region of the abdomen was sheared, shaved and disinfected using 10% polyvinylpyrrolidone iodine (AGB, Madrid, Spain). A 2 cm incision was made in the skin over the midline and subcutaneous tissue was dissected. The white line and peritoneum were sectioned with blunt scissors to avoid haemorrhage. A 40 cm long cannula connected to a syringe was inserted into the abdominal cavity and 40 ml sterile DPBS containing 9500 UI of heparin (Eurotubo®, Madrid, Spain) (warmed at 37 °C) was injected into the abdominal cavity. After softly massaging the abdominal cavity for 1 min, 40 ml of peritoneal fluid were withdrawn. Peritoneal fluid was centrifuged at 1500× *rpm* for 5 min and the supernatant was discarded. Cell pellets were resuspended again in DPBS and incubated for 15 min in an erythrolysis buffer (0.15 M NH_4_Cl, 10 mM KHCO_3_, 0.1 mM disodium EDTA, dH_2_O). A second centrifugation step (1500× *rpm* for 5 min) was performed to eliminate lysed erythrocyte membranes and the pellet was resuspended in 1 ml of medium. After that, the concentration of peritoneal cells was quantified using the Trypan Blue exclusion technique. The final concentration was adjusted to 1 × 10^6^ cells/ml for analysis by flow cytometry assay.

### Flow cytometry assay

Flow cytometry acquisition was performed with a CyFlow Cube 6® (Sysmex-Partec, Barcelona, Spain) cytometer. Leukocytes were identified by their characteristic appearance on a FSC-SSC dot plot and gated in order to exclude cellular debris. Ten thousand events were analysed for changes in fluorescence intensity (enzymatic activity) of macrophages and granulocytes. For oxidative metabolism, hydrogen peroxide (H_2_O_2_) and nitric oxide (NO) production, filtered light at green was used (488 nm FL1 channel). The results were analysed using FCS Express 4.0 (DeNovo Software, Los Angeles, USA) and Flowing Software 2.0 (Centre for Biotechnology, Turku, Finland).

In order to measure intracellular H_2_O_2_, the cell-permeable dye DCFH-DA (Dichloro-dihydro-fluorescein diacetate) was used. The non-fluorescence reduced form is converted into the fluorescent form when is oxidised and, in this way, the fluorescence can be detected by flow cytometry. A 200 μl sample of peritoneal cells were incubated in dark and 37 °C for 20 min with 1 ml of DCFH-DA 10 μM. After incubation, samples were centrifuged at 1500× *rpm* for 5 min and the supernatant was discarded. Two washes of PBS centrifuged at 1500× *rpm* for 5 min were carried out and the pellets are resuspended in DPBS.

Intracellular NO was measured using the cell-permeable dye DAF-2DA (4,5-diaminofluorescein diacetate), because the non-fluorescent reduced form is converted into the fluorescent form when oxidised, thus allowing the detection by flow cytometry. In the same way, a 200 μl sample of peritoneal cells was incubated in dark and 37 °C for 180 min. From this point onwards the methodology was similar to that described for DCFH-DA.

### Statistical analysis

Statistical analysis was carried out with GraphPad Prism v.6.0 (GraphPad Software Inc., San Diego, CA, USA). The Kolmogorov-Smirnov test was applied to evaluate whether distributions were parametric. Comparison between pairs of groups was made using a two-tailed Mann-Whitney U-test for non-parametric distributions. *P* < 0.05 was considered statistically significant.

## Results

### Progression of infection: liver pathology and antibody production

No hepatic changes were observed in the negative control animals (Group 1). In infected and vaccinated animals (Groups 2 and 3), gross and histopathological hepatic changes were absent at 1 and 3 dpi, though tortuous whitish tracts and haemorrhagic spots, which mainly occurred along the left hepatic lobe, were detected at 9 and 18 dpi. Further description of the gross hepatic lesions as well as the histopathological changes have been previously described in our recent study [18].

**Fig. 1:**
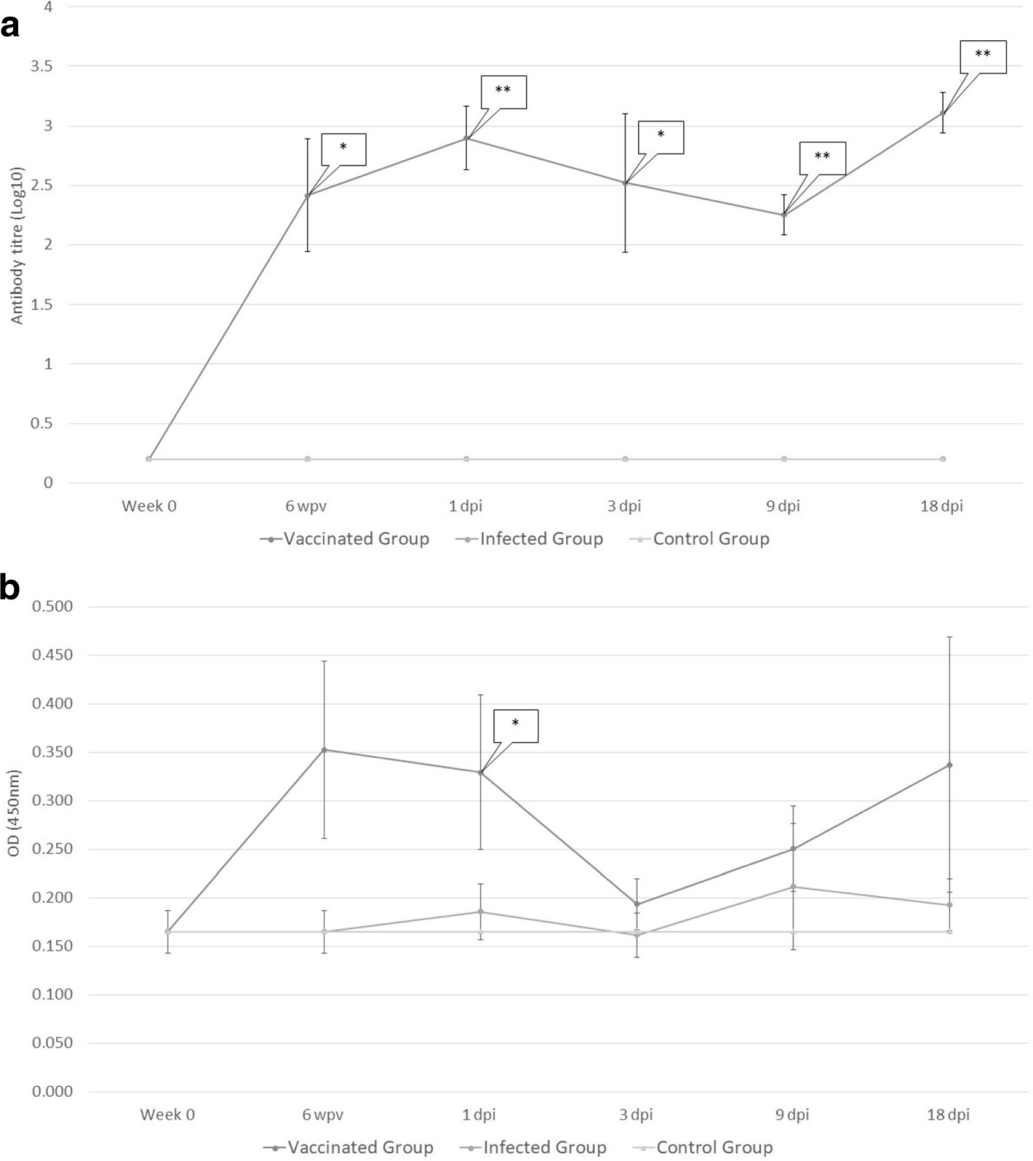
Plasma levels of specific rFhCL1 IgG1 (**a**) and IgG2 (**b**). Each point represents mean values of antibody titre - log10 - (IgG1) and of optical density (IgG2) measured at 450 nm. Bars at each point represents tandard error. Immunisation with rFhCL1 developed a significant rise in the level of IgG1 isotype; dynamics of IgG1 in uninfected and infected sheep (Groups 1 and 2) shows a similar pattern, hence it is overlapped in the figure. Significant IgG2 production was detected only at 1 day post-infection (dpi) during the trial

The levels of plasmatic specific anti-rFhCL1 IgG1 and IgG2 are presented in Fig. 1a, b. All vaccinated animals with the recombinant antigen developed an IgG1-IgG2 antibody response following immunisation. In vaccinated animals, a statistically significant production of IgG1 was detected after immunisation at each slaughtering time-point (*U* = 0, *df* = 8, *P* = 0.0006 for 6 wpv; *U* = 0, *df* = 8, *P* = 0.0097 for 1, 9 and 18 dpi and *U* = 3, *df* = 8, *P* = 0.0449 for 3 dpi) and showing an increasing trend at the end of the trial (Fig. 1a). Production of IgG2 showed a similar pattern to that observed for IgG1 but was overall limited and only statistically significant after immunisation (*U* = 2, *df* = 8, *P* = 0.0062; Fig. 1b). No production of specific anti-rFhCL1 IgG1 and IgG2 was detected in the negative and positive control groups (Groups 1 and 2).

### Dynamics of peritoneal leukocytes during infection

**Fig. 2:**
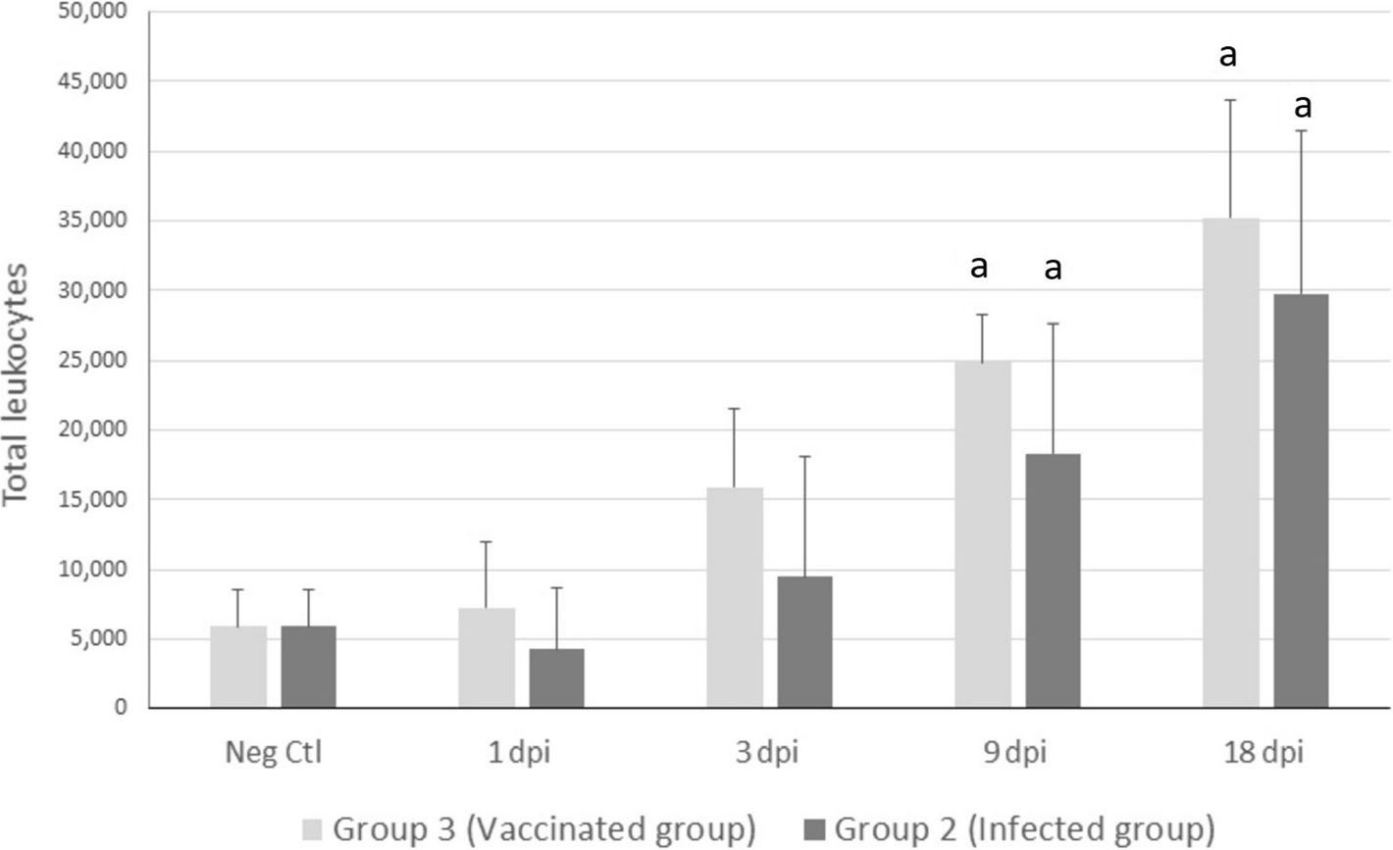
Mean total number of leukocytes illustrating the effect of infection on peritoneal cell recruitment. Cell viability was assessed by trypan blue exclusion. “a” indicates significant variations between infected (Group 2, infected group), immunised (Group 3, vaccinated group) and control (Neg Ctl) sheep (*P* < 0.05) at 4 slaughtering time-points: 1, 3, 9 and 18 days post-infection (dpi)

The total number of leukocytes population from peritoneal liquid was assessed by flow cytometry and expressed as 1 × 10^6^ cells/ml. There was a significant increase in the number of leukocytes at 9 and 18 dpi, in both infected (*U* = 3, *df* = 8, *P* = 0.0439 and *U* = 0, *df* = 8, *P* < 0.0001, respectively) and vaccinated (*U* = 0, *df* = 8, *P* = 0.0009 and *U* = 0, *df* = 8, *P* < 0.0001, respectively) groups in comparison with the negative control group (Fig. 2).

### Distribution of peritoneal leukocyte populations during infection

**Fig. 3:**
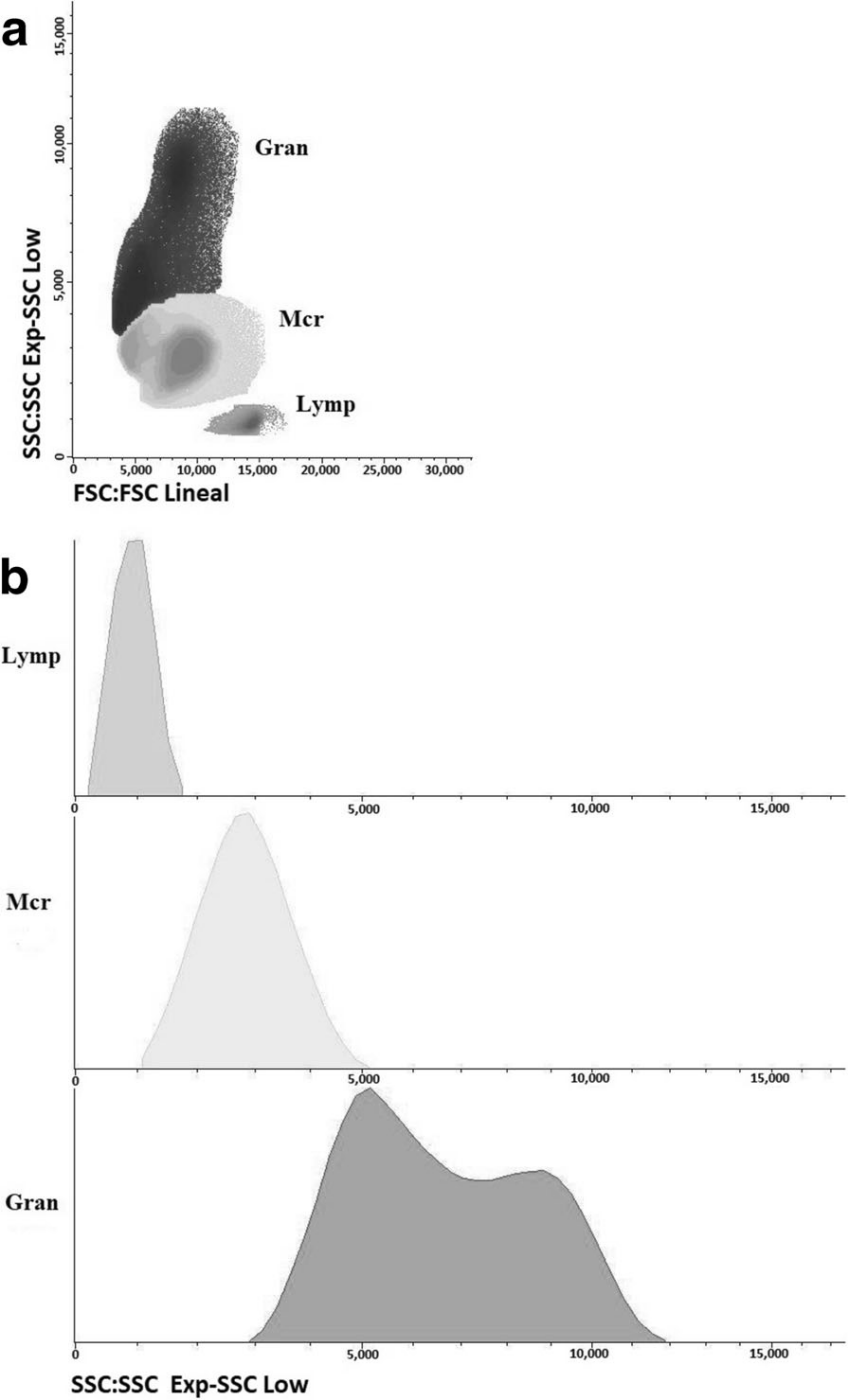
Flow cytometry analysis of peritoneal cell population of sheep after peritoneal lavage characterised by a side scatter/forward scatter (SSC/FSC) profile. Distribution of major leukocyte populations on gated regions is represented by dot-plot (**a**) and histogram (**b**). *Abbreviations*: Lymp, lymphocytes; Mcr, macrophages; Gran, granulocytes

**Fig. 4:**
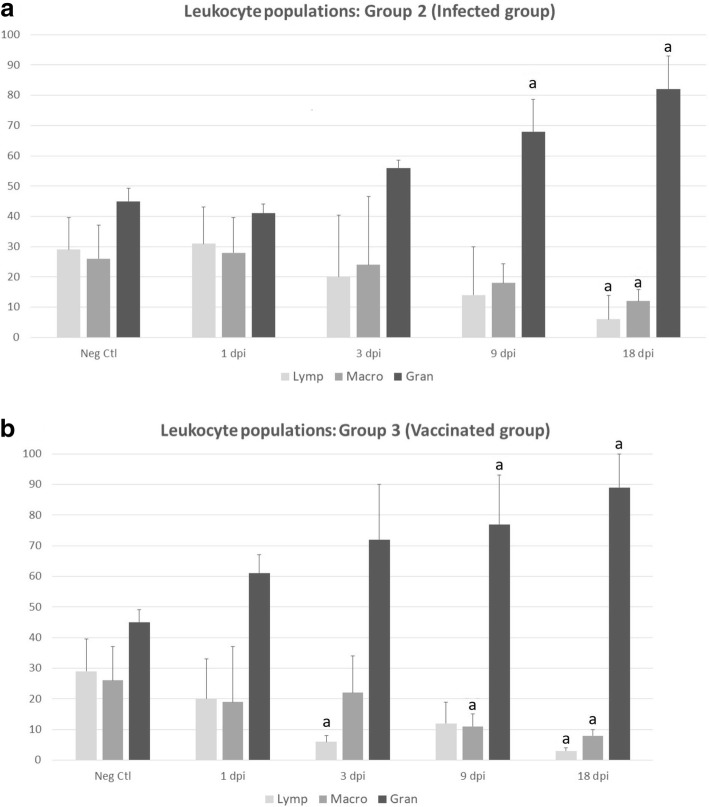
Differential cell counts by flow cytometric analysis of peritoneal lavage leukocyte samples from uninfected (Neg Ctl, Group 1, **a**, **b**), infected (Group 2, **a**) and vaccinated sheep (Group 3, **b**). Each identified cell subset is expressed as a percentage of the total number of leukocytes. Values represent the mean ± SD. “a” indicates significant differences (*P* < 0.05) between groups. *Abbreviations*: Lymp, lymphocytes; Macro, macrophages; Gran, granulocytes

Leukocyte populations were characterised by a side scatter/forward scatter profile as shown in Fig. 3a, b. Dynamics of cell populations through the infection is shown in Fig. 4a, b. The trend in all animals was an increase in the number of granulocytes and a decrease in both macrophages and lymphocytes throughout the infection.

Granulocytes were significantly increased in the infected group at 9 dpi (*U* = 0, *df* = 8, *P* = 0.0129) and 18 dpi (*U* = 2, *df* = 8, *P* = 0.0032). In the vaccinated group, a similar significant increment was detected at 9 dpi (*U* = 3, *df* = 8, *P* = 0.0481) and at 18 dpi (*U* = 0, *df* = 8, *P* = 0.0071). On the other hand, a significant decrease in the number of lymphocytes (*U* = 3, *df* = 8, *P* = 0.0098) and macrophages (*U* = 6, *df* = 8, *P* = 0.0291) was observed in the infected group at 18 dpi compared with the negative control group (Group 1).

Lymphocytes and macrophages were significantly decreased in vaccinated animals (*U* = 0, *df* = 8, *P* = 0.0011 and *U* = 1, *df* = 8, *P* = 0.0058, respectively) and in infected group (*U* = 3, *df* = 8, *P* = 0.0098 and *U* = 6, *df* = 8, *P* = 0.0291, respectively) at 18 dpi compared to negative control animals (Group 1). This decrease was also observed in the vaccinated animals at 3 dpi for lymphocytes (*U* = 6, *df* = 8, *P* = 0.0037) and at 9 dpi for macrophages (*U* = 2, *df* = 8, *P* = 0.0182), compared to the negative control group.

### Hydrogen peroxide production by peritoneal leukocyte populations during infection

**Fig. 5:**
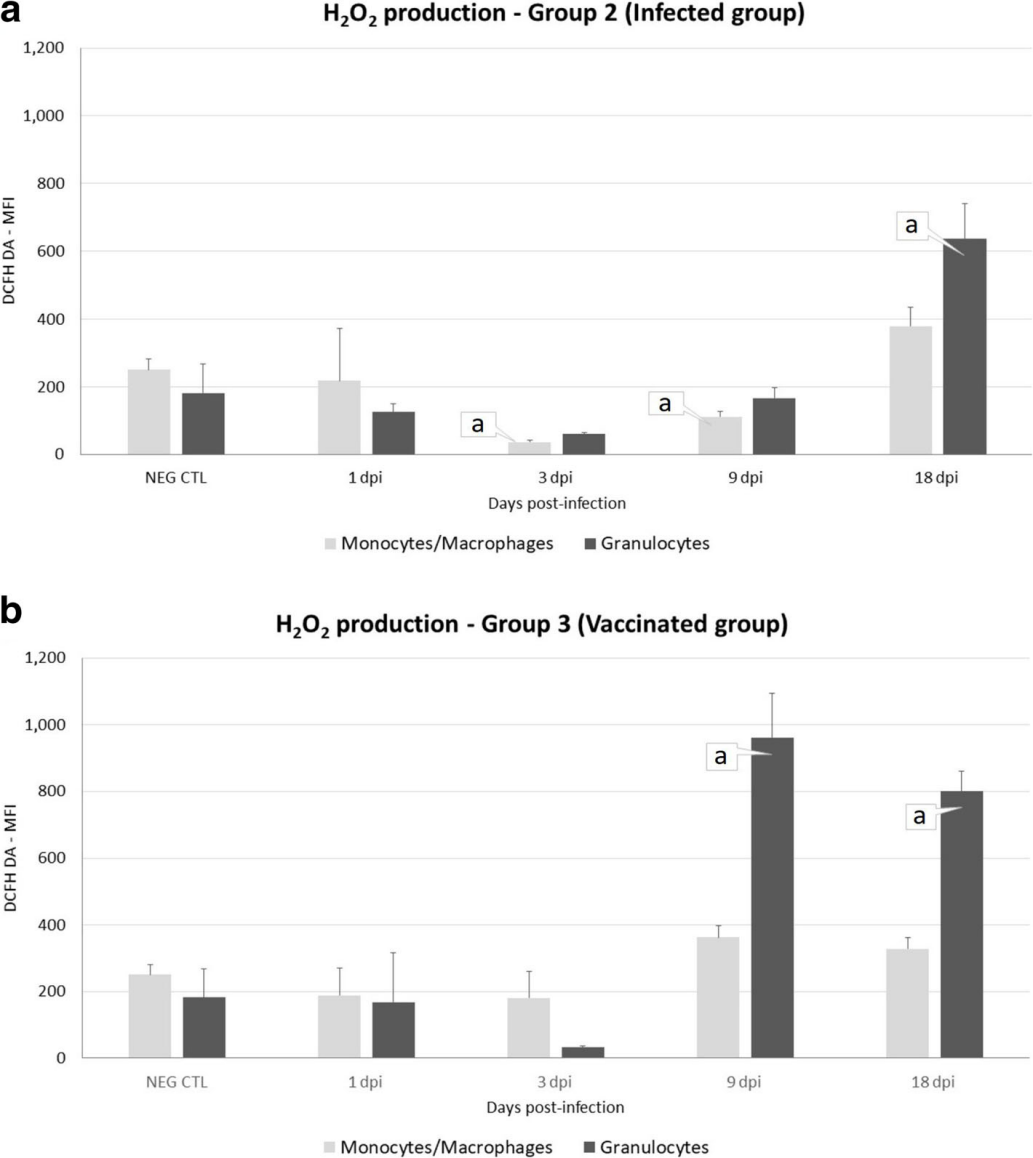
Mean fluorescence intensity of H_2_O_2_ production (DCFH DA) in the peritoneal fluid of uninfected sheep (Neg. Ctl, Group 1, **a**, **b**), infected (Group 2, **a**) and vaccinated sheep (Group 3, **b**). Values represent the mean ± standard deviation, SD. “a” indicates significant differences (*P* < 0.05) between groups

The results are shown in Fig. 5a, b. Production of H_2_O_2_ displayed a slightly different overall pattern in infected and vaccinated animals. During the initial stage of the infection, H_2_O_2_ production occurred mainly in the granulocyte cell type. In the infected group (Fig. 5a), the increase of H_2_O_2_ production by granulocytes was only observed at 18 dpi (*U* = 2, *df* = 8, *P* = 0.0002). Nevertheless, a significant decrease was observed at 3 dpi for monocytes/macrophages (*U* = 0, *df* = 8, *P* = 0.0296).

In vaccinated animals (Fig. 5b) granulocytes showed a significant increase (*U* = 0, *df* = 8, *P* < 0.0001) in H_2_O_2_ production at 9 and 18 dpi, whereas no significant variations were observed in the response of monocytes/macrophages.

A statistically significant higher level of H_2_O_2_ production by granulocytes was observed in the vaccinated group than in the infected group at 9 dpi (*U* = 0, *df* = 8, *P* < 0.0001).

### Nitric oxide production by peritoneal leukocyte populations during infection

**Fig. 6:**
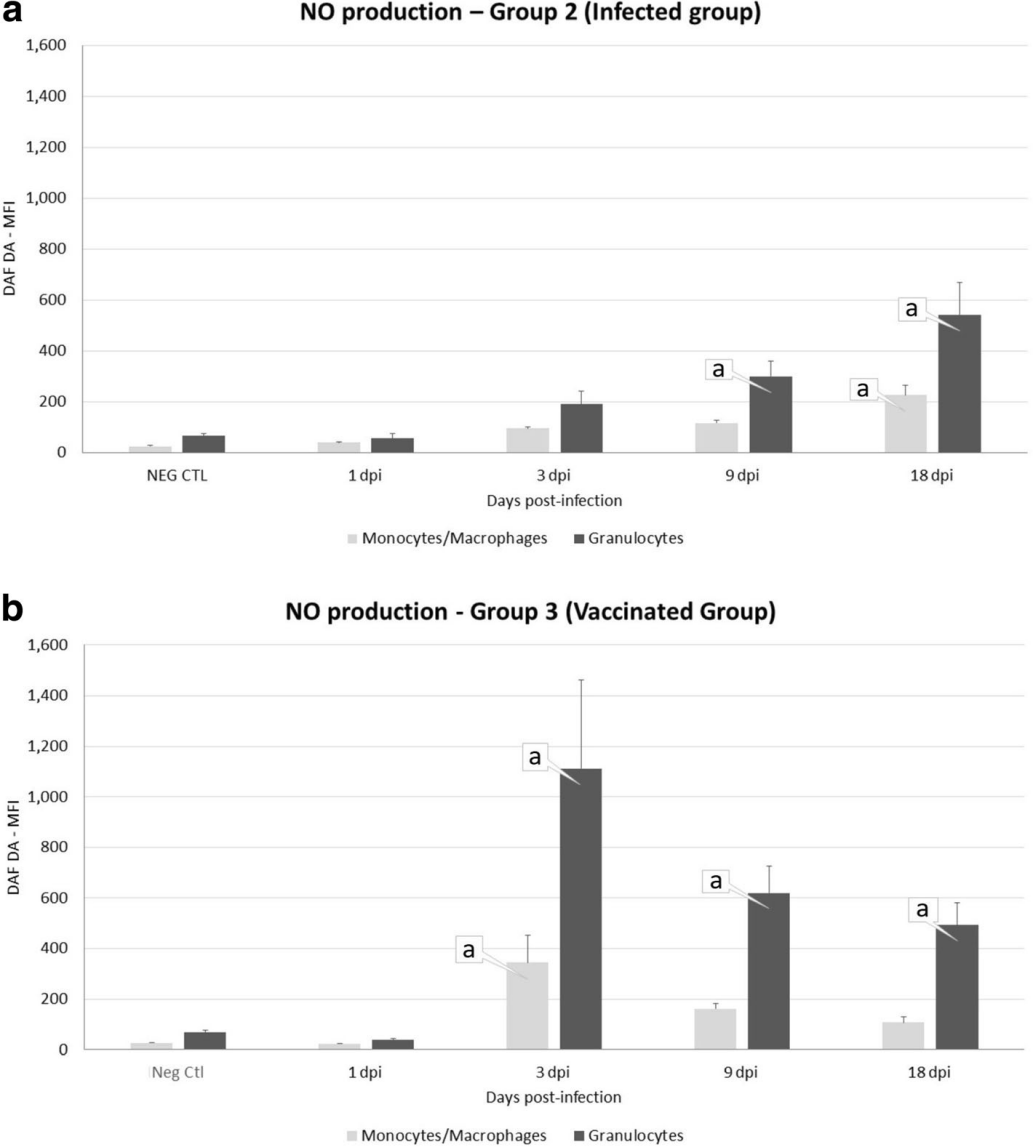
Mean fluorescence intensity of NO production (DAF DA) in the peritoneal fluid of sheep uninfected (Neg Ctl, Group 1, **a**, **b**), infected (Group 2, **a**) and vaccinated sheep (Group 3, **b**). Values represent the mean ± standard deviation, SD. “a” indicates significant differences (*P* < 0.05) between groups

The dynamic of NO production is shown in Fig. 6a, b. In the infected animals (Fig. 6a) a significant rise in NO production was detected by granulocytes at 9 dpi (*U* = 0, *df* = 8, *P* = 0.0432) and 18 dpi (*U* = 0, *df* = 8, *P* = 0.0076), and by monocytes/macrophages at 18 dpi (*U* = 0, *df* = 8, *P* = 0.0022). In vaccinated sheep (Fig. 6b) the NO production by granulocytes was statistically significant increased at 3 dpi (*U* = 0, *df* = 8, *P* < 0.0001), 9 dpi (*U* = 0, *df* = 8, *P* = 0.0026) and 18 dpi (*U* = 0, *df* = 8, *P* = 0.0133), whereas production by monocytes/macrophages was significantly increased only at 3 dpi (*U* = 0, *df* = 8, *P* < 0.0001).

Production of NO by granulocytes was statistically significant higher at 3 dpi (*U* = 0, *df* = 8, *P* < 0.0001) and 9 dpi (*U* = 3, *df* = 8, *P* = 0.0476) in the vaccinated group than in the infected group.

## Discussion

This study focuses on the production of free radicals by leukocyte populations in peritoneal fluid during the early stages of fasciolosis, being the first *in vivo* study carried out in sheep infected and uninfected with *F. hepatica*. We have compared the response in sheep vaccinated with rFhCL1 and non-vaccinated animals and we have confirmed the existence of a noticeable NO response, mainly by granulocytes, in both infected and vaccinated animals.

The peritoneal cavity is a critical location in the development of *F. hepatica* infection as it is the route of migration of *F. hepatica* NEJ from the intestine to the liver and the site where an early immunomodulatory effect of the parasite likely plays a critical role in determining the ultimate outcome of the infection [9]. It has been suggested that effector mechanisms of NEJ killing and consequent immune protection are dependent on the activity of peritoneal leukocytes, as described for *F. hepatica* in rats [19] and *F. gigantica* in Indonesian thin-tail sheep [15]. Both antibodies and free radicals (ROS and NO) produced by leukocytes have been considered effective elements in those protective peritoneal responses [14]. However, ROS and NO produced as a strategy to kill parasites during *F. hepatica* infection have been also described as responsible for oxidative stress and hepatic damage in sheep [20], rats [21] and cattle [22].

In our experiment, peritoneal leukocyte populations in sheep increased immediately after infection, reaching significant levels at 9 and 18 dpi in both infected and vaccinated groups, as previously described in rats experimentally challenged with *F. hepatica* [14]. From 9 dpi onwards, hepatic lesions could be detected, associated with the penetration of NEJ in the liver parenchyma. The cellular infiltration in hepatic lesions was mainly composed of eosinophils, macrophages and lymphocytes, whereas the peritoneal populations were mainly granulocytes, in increased proportions over the course of the infection. In our study, by using the flow cytometry assay, we could not discriminate the different cell population of granulocytes (neutrophils, eosinophils and basophils), but using immunocytochemistry staining, we have determined that eosinophils occurred in more than 95% of total peritoneal granulocyte populations (data not shown) [23]. The predominance of granulocytes and more specifically of eosinophils in the peritoneal cell populations have been also observed in the early stage of infection in rats [11, 24], in goats by immunohistochemical studies [17, 25] and in sheep by transcriptome analysis [26].

By means of the flow cytometry technique, we could assess the intracellular production of free radicals (H_2_O_2_and NO) by peritoneal macrophages and granulocytes, although we could not identify the different oxidative response of neutrophils and eosinophils. The intracellular production of both H_2_O_2_ and NO by peritoneal leukocytes was stimulated during the early stages of infection, in the infected group and in the vaccinated group, as previously described in rats [14]. However, these authors found macrophages as the most significant cell type at the initial stage of the infection (7 dpi) in contrast to our findings in sheep, in which granulocytes were proved to be more relevant cells at all time-points of the study.

We have also found that macrophages and particularly granulocytes from vaccinated animals showed a significantly higher production of free radicals, mainly at 9 and 18 dpi. This is consistent with our previous work where a partial protective response was described in experimental trials with rFhCL1, that could be related to eosinophils and free radical (NO) production in the early stage of infection [17, 18].

The role of H_2_O_2_ in *F. hepatica* infection remains unclear. Transcriptome studies in mice revealed production of ROS as one the most significant pathways undergoing changes during immunoprotection [27] but it has also been related to oxidative stress and pathology in chronic infection in sheep [20]. Moreover, different *in vitro* studies indicate that *F. hepatica* NEJ possess a unique ability to resist killing by reactive oxygen species released by sheep innate immune effector cells, which may involve the high expression of antioxidant enzymes such as superoxide dismutase, glutathione S-transferase (GST) or peroxiredoxin [28–31].

In our study, both macrophages and granulocytes were involved in the increase of NO production occurring between 3 and 18 dpi. Previous studies in rats have highlighted the involvement and complementary role of these two cell populations in the peritoneal stage of the infection [11, 14, 31]. In fact, Piedrafita et al. [15] described an antibody-dependent cell-mediated killing of NEJ involving both macrophages and eosinophils NO production in the resistance of ITT sheep to *F. gigantica*. Our previous vaccination study suggested that inducible nitric oxide synthase (iNOS) expression and subsequent NO production could be important for an effective response against the early migrating liver fluke [17]. Although the early production of NO we have detected in that study seemed to have little effect on the development of the infection and in the final fluke burden at the end of the experiment [18]. Consistent protective responses in sheep has not been achieved and the precise effective mechanisms of protection has not been yet elucidated [9]. It has been hypothesised that NO and iNOS might play an important role in *F. hepatica* pathogenesis, possibly as an effective mechanism for killing migrating NEJ, as it has been previously shown to occur in resistant rats [31] or maybe as an expression of M1 macrophages activation which are known to be related to the development of Th1 responses required for protection [18, 32, 33]. Recent transcriptomic studies have revealed that modifications in the NO signalling pathway may be a necessary condition for immunoprotection in mice [27] or, on the contrary, downregulation of iNOS might be a paramount factor during the non-protective response occurring in sheep [34]. In our study, we have not detected an inhibition in NO production in the early phase of infection. In another study, we found a low level of variation in iNOS expression in peritoneal macrophages by immunocytochemistry [35]. Those differences suggest that iNOS gene, protein expression and NO production in the initial stages of the infection may differ, with the protein probably remaining active for a longer time than the gene.

In conclusion, we have observed a clear leukocyte response in the peritoneal cavity of the sheep in the early stage of *F. hepatica* infection. The leukocyte populations, mainly granulocytes, exhibited a metabolic response with intracellular production of both H_2_O_2_ and NO. The effect of those free radicals on the NEJ and migrating juveniles it is still unclear, since NEJ and migrating juveniles appear to be unaffected by those molecules as they reached the liver and evolved to mature stages. Further studies are needed to provide a broader insight on the biomolecular mechanisms involved in the evasion of the immune response of this parasite in the early stages of infection.

## Conclusions

Experimental infection induced an increase in the total number of leukocytes within the abdominal cavity at 9 and 18 dpi which was characterised by an increase in the number of granulocytes and a decrease of both macrophages and lymphocytes. Production of both H_2_O_2_ and NO by peritoneal cells was increased in vaccinated and non-vaccinated animals. Granulocytes were mainly involved in H_2_O_2_ production, whereas granulocytes and macrophages were predominant in NO production. Vaccinated animals produced a significantly higher level of H_2_O_2_ and NO than infected animals.

## Abbreviations

dpi: days post-infection
GST: glutathione S-transferase
H_2_O_2_: hydrogen peroxide
iNOS: inducible nitric oxide synthase
NEJ: newly excysted juveniles
NO: nitric oxide
rFhCL1: recombinant cathepsin L1 of *Fasciola hepatica*
ROS: reactive oxygen species
TCBZ: triclabendazole
wpv: weeks post-vaccination
